# Lung function in adults born preterm

**DOI:** 10.1371/journal.pone.0205979

**Published:** 2018-10-19

**Authors:** Pieta Näsänen-Gilmore, Marika Sipola-Leppänen, Marjaana Tikanmäki, Hanna-Maria Matinolli, Johan G. Eriksson, Marjo-Riitta Järvelin, Marja Vääräsmäki, Petteri Hovi, Eero Kajantie

**Affiliations:** 1 National Institute for Health and Welfare, Helsinki and Oulu, Finland; 2 Institute of Health Sciences, University of Oulu, Oulu, Finland; 3 PEDEGO Research Unit, Medical Research Centre Oulu, Oulu University Hospital and University of Oulu, Oulu, Finland; 4 Folkhälsan Research Centre, Helsinki, Finland; 5 Department of General Practice and Primary Health Care, University of Helsinki, Helsinki, Finland; 6 Vaasa Central Hospital, Vaasa, Finland; 7 MRC Health Protection Agency (HPA) Centre for Environment and Health, School of Public Health, Imperial College London, London, United Kingdom; 8 Biocenter Oulu, University of Oulu, Oulu, Finland; 9 Unit of Primary Care, Oulu University Hospital, Oulu, Finland; 10 Children’s Hospital, Helsinki University Central Hospital, Helsinki, Finland; 11 Department of Clinical and Molecular Medicine, Norwegian University of Health and Technology, Trondheim, Norway; Univesity of Iowa, UNITED STATES

## Abstract

Very preterm birth, before the gestational age (GA) of 32 weeks, increases the risk of obstructed airflow in adulthood. We examined whether all preterm births (GA<37 weeks) are associated with poorer adult lung function and whether any associations are explained by maternal, early life/neonatal, or current life factors. Participants of the ESTER Preterm Birth Study, born between 1985 and 1989 (during the pre-surfactant era), at the age of 23 years participated in a clinical study in which they performed spirometry and provided detailed medical history. Of the participants, 139 were born early preterm (GA<34 weeks), 239 late preterm (GA: 34-<37 weeks), and 341 full-term (GA≥37 weeks). Preterm birth was associated with poorer lung function. Mean differences between individuals born early preterm versus full-term were -0.23 standard deviation (SD) (95% confidence interval (CI): -0.40, -0.05)) for forced vital capacity z-score (zFVC), -0.44 SD (95% CI -0.64, -0.25) for forced expiratory volume z-score (zFEV1), and -0.29 SD (95% CI -0.47, -0.10) for zFEV1/FVC. For late preterm, mean differences with full-term controls were -0.02 SD (95% CI -0.17, 0.13), -0.12 SD (95% CI -0.29, 0.04) and -0.13 SD (95% CI -0.29, 0.02) for zFVC, zFEV1, and zFEV1/FVC, respectively. Examination of finer GA subgroups suggested an inverse non-linear association between lung function and GA, with the greatest impact on zFEV1 for those born extremely preterm. The subgroup means were GA<28 weeks: -0.98 SD; 28-<32 weeks: -0.29 SD; 32-<34 weeks: -0.44 SD; 34-<36 weeks: -0.10 SD; 36-<37weeks: -0.11 SD; term-born controls (≥37weeks): 0.02 SD. Corresponding means for zFEV1/FVC were -1.79, -0.44, -0.47, -0.48, -0.29, and -0.02. Adjustment for maternal pregnancy conditions and socioeconomic and lifestyle factors had no major impact on the relationship. Preterm birth is associated with airflow limitation in adult life. The association appears to be attributable predominantly to those born most immature, with only a modest decrease among those born preterm at later gestational ages.

## Introduction

Preterm birth, or birth before 37 gestational weeks, accounts for approximately 11% of all births globally: annually ~15 million births worldwide [1]. In the United States, as an example, the proportion of premature births increased from 9.5% to 12.7% between 1998 and 2005 [2], with a modest decrease thereafter. Recent improvements in perinatal and neonatal care have led to increased survival of preterm-born individuals worldwide.

Children and adolescents born preterm have more airflow obstruction and manifest more airway disorders [3–8] than their peers born at term. However, research on long-term lung function outcomes has primarily focused on those born very preterm at gestational age (GA) less than 32 weeks or with very low (<1500 grams) or extremely low (<1000 grams) birth weight [9–12]. These individuals together constitute 1–2% of all live births, and they often require mechanical ventilation and are more likely to have bronchopulmonary dysplasia (chronic lung disease of prematurity) at a rate of approximately 10–40%, depending on definition [13]. However, relatively little is known about the lung health of the much larger group of adults born moderately or late preterm (GA: 32-<34weeks or GA: 34-<37 weeks resprectively) [14]. While bronchopulmonary dysplasia in these groups is rare (or, according to some definitions, cannot occur), these groups still are at risk of airflow limitation in childhood, which could persist to adult life [5]. Furthermore, maternal pregnancy disorders (hypertensive disorders, gestational diabetes) and other risk factors of preterm birth, including maternal gestational smoking, are likely to influence pulmonary development [4,15–18]. It remains unclear to what extent these intrauterine conditions contribute to the association between preterm birth and adult lung function.

Our aim was to assess lung function and pulmonary health in adults born preterm across the whole gestational age range. We hypothesized an inverse relationship between gestational age at birth and airflow impairment.We also explored whether potential associations with preterm birth may be attributed to neonatal conditions or to maternal pregnancy conditions underlying preterm birth.

## Methods

### Subjects

**Fig 1:**
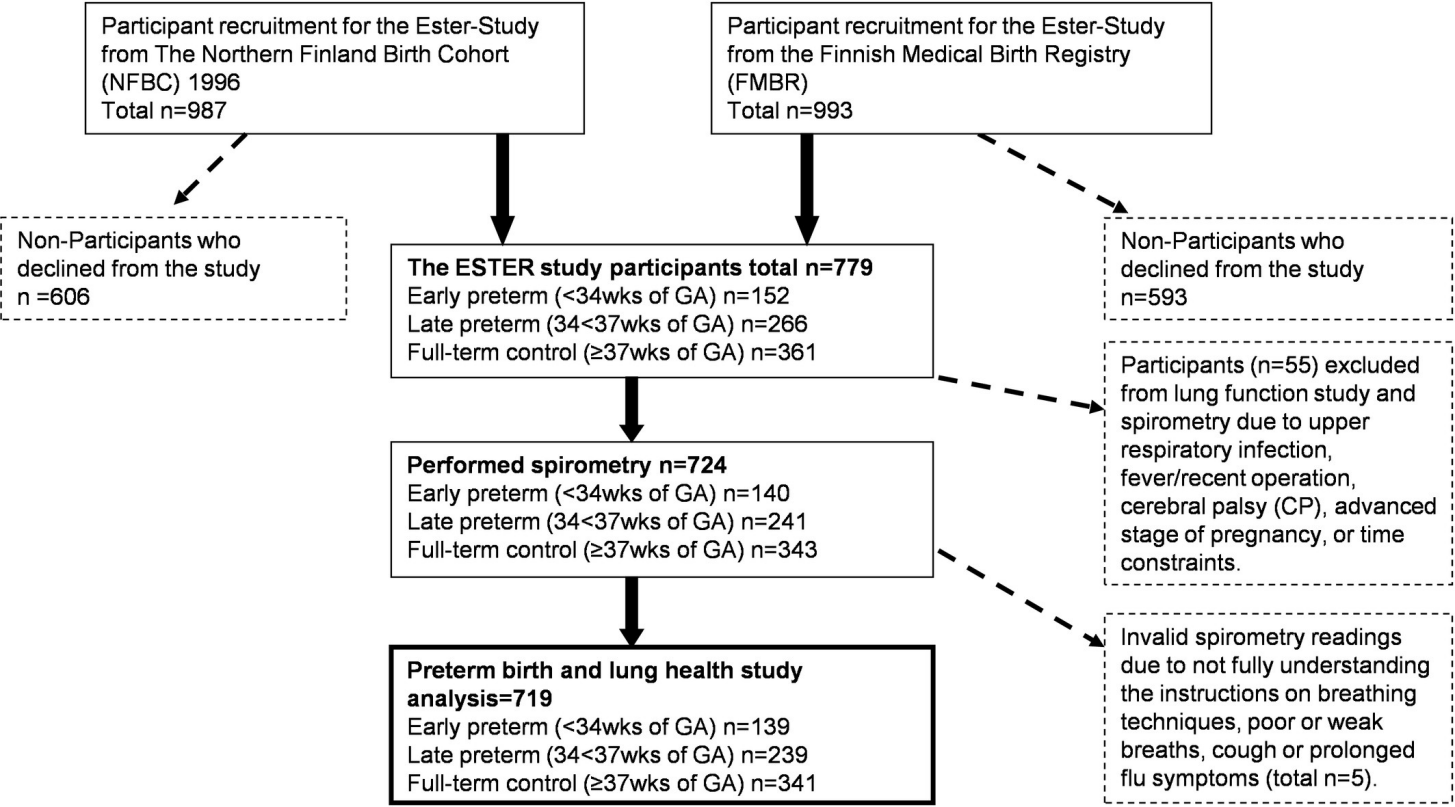
Participant recruitment process.

Participants come from the ESTER Preterm Birth Study, which comprises 1980 young adults. They were grouped by gestational age at birth into those born early preterm (GA<34 weeks), late preterm (GA 34-<37 weeks) and full-term (GA≥37 weeks) as the control group, which we refer to as ‘at term’ (Fig 1). Participantswere recruited among the 1986 Northern Finland Birth Cohort (NFBC), born from 1985 to 1986 (49.8%), and among all children (50.2%) born from 1987 to 1989 within the same geographical area; they were identified through the Finnish Medical Birth Register (FMBR) [19]. The births preceded the surfactant era. According to a previously published detailed non-participant analysis [19] of the clinical examination, among the late preterm and control groups, men were less likely to participate; there were no differences in perinatal characteristics between participants and non-participants in any of the GA groups.

### Measurements

From 2009 to 2011, 779 individuals with adequately verified lengths of gestation participated in a clinical study at a mean age of 23.3 (standard deviations, SD 1.2) years and underwent a thorough health examination, including spirometry; they reported on pulmonary health, physical activity, and smoking as well as family socio-demographics [19]. Of the participants, 724 performed spirometry (detailed methods described in the Protocol below). After excluding individuals unable to perform spirometry reliably, 139 participants born early preterm, 239 born late preterm, and 341 born at term were included in the analyses of this paper.

We obtained details on pregnancy, birth and postnatal period from medical records (S1 Table), calculated birth weight z-scores [20], and independently verified the length of gestation, which was based on ultrasound (preterm births: 56.8%, controls: 43.2%) or last menstrual period [21]. We verified diagnoses of gestational diabetes, gestational or chronic hypertension, and pre-eclampsia or super-imposed pre-eclampsia according to prevailing medical guidelines [22,23]. Bronchopulmonary dysplasia (BPD) was identified in two ways: by pediatrician’s diagnosis (for NFBC), or by the need for supplementary oxygen or ventilator support at 28 days of age (for FMBR) [24].

### Protocol

Spirometry (n = 724) was performed (Medikro Windows, Spiro 20001.3) sitting upright following the standard spirometry technique described by the European Respiratory Society Guidelines [25]. The same testing techniques were applied to all participants. Lung function parameters were recorded as follows: forced vital capacity (FVC), forced expiratory volume in 1 second (FEV1), ratio of FEV1/FVC, peak expiratory flow (PEF), forced midexpiratory flow (FEF_25-75%_), forced expiratory flow at 75% of expired volume during FVC test (FEF_75%_), forced expiratory flow at 50% of expired volume during FVC test (FEF_50%_). Lung function data were converted into Global Lung Function Initiative z-scores using the Caucasian reference [26]. Data were also described as percentage of predicted, following the Finnish standards [27], based on the American Thoracic Society and European Respiratory Societies’(ATS/ERS) guidelines [28].

Bronchodilation test was performed [25], according to Finnish guidelines, when the baseline readings indicated airway obstruction as follows: FVC<80%, FEV1<90%, FEV1/FVC<88%, PEF<74%, FEF50%<62%, or FEF75%<48% [25, 29]. Participants inhaled bronchodilator (400 μg salbutamol) and repeated spirometry 10 to 15 minutes thereafter. As per the guidelines, a minimum of 12% and 0.2 l improvement in FVC or FEV1 was considered a positive bronchodilation test [25, 27, 30]. This criterion of 12% and 0.2 l improvement in FVC or FEV1 is based on the Finnish bronchodilation test guideline [29–30] and ATS/ERS guidelines [28].

### Data analyses

All statistical analyses (univariate analysis of variance [ANOVA], Chi-square test, and Fisher’s exact test and regression) were preformed using SPSS v.22 (SPSS Inc, Chicago, US). Lung function, as the main outcome of interest, was compared between the preterm groups and the term-born controls as a continuous outcome by multiple linear regression. We also compared the proportion of participants with low zFVC, zFEV1 or zFEV1/FVC, defined as being below -1.645 SD, corresponding to 5^th^ percentile, by multiple logistic regression. We used two multi-variable regression models, adjusting for covariates related to intrauterine exposures (maternal hypertensive disorders, gestational diabetes, smoking, and intrauterine growth restriction), parental education, and participant health (detailed descriptions of covariates are provided in S1 Table. Categorical variables were dummy-coded for the purpose of analyses. Participants’ background details per GA groups are provided in Table 1. S2 Table shows descriptive data on lung function and lung health variables for those born early and late preterm and for term-born controls.

**Table 1:** Study background characteristics of the ESTER birth cohort (births in Northern Finland during 1985–89): Clinical Examination during 2009–11.

Variable	Subgroup	Early Preterm <34 weeks	Late preterm 34-<37 weeks	Full-term ≥37weeks	Missing (n)
		n = 139	n = 239	n = 341	Early pretem/Late Preterm /Term
		mean (SD)/n (%)	mean (SD)/n (%)	mean (SD)/n (%)	
Maternal smoking during pregnancy ^a^		23 (16.5%)	46 (19.2%)	54 (15.8%)	0/0/0
Highest parental education ^a^	Secondary or less or unknown	97 (69.8%)	159 (66.5%)	229 (67.2%)	0/0/0
	Lower tertiary	13 (9.4%)	30 (12.6%)	44 (12.9%)	
	Upper tertiary	29 (20.9%)	50 (20.9%)	68 (19.9%)	
Maternal body mass index before pregnancy ^b^		22.5 (3.5)	22.7 (4.0)	22.3 (3.0)	8/7/13
Antenatal glucocorticoid treatment^c^		20 (22.2%)	7 (6.5%)	0 (0%)	0/0/0
Maternal gestational diabetes^d^		4 (2.9%)	11 (4.6%)	6 (1.8%)	0/0/0
Hypertensive disorder during pregnancy^**†**^	Normotensive^a^	85 (63.0%)	167 (72.3%)	274 (84.3%)	4/8/16
	Gestational hypertension^a^	7 (5.2%)	20 (8.7%)	27 (8.3%)	
	Preeclampsia^a^^**†**^	23 (17.0%)	22 (9.5%)	8 (2.5%)	
	Chronic hypertension**	12 (8.9%)	16 (6.9%)	9 (2.8%)	
	Superimposed preeclampsia*	8 (5.9%)	6 (2.6%)	7 (2.2%)	
Mean length of gestation (weeks)^b^^**†**^		31.8 (1.9)	35.8 (0.8)	40.1 (1.2)	0/0/0
Maternal age at birth^b^		29.4 (5.4)	29.4 (6.0)	28.1 (5.5)	0/0/0
Multiple pregnancy (twins)^d^		33 (23.7%)	32 (13.4%)	4 (1.2%)	0/0/0
Source cohort^a^^,^^e^^**†**^	NFBC	49 (35.3%)	131 (54.8%)	205 (60.1%)	0/0/0
	FMBR	90 (64.7%)	108 (45.2%)	136 (39.9%)	0/0/0
Sex^a^	Male	66 (47.5%)	118 (49.4%)	164 (48.1%)	0/0/0
	Female	73 (52.5%)	121 (50.6%)	177 (51.9%)	
Birth weight (g)^b^^**†**^		1777 (482)	2670 (521)	3583 (485)	0/0/0
Birth weight SD score^b^^**†**^		-0.72 (1.40)	-0.64 (1.29)	-0.00 (1.00)	0/0/0
SGA (birth weight<-2SD)^a^^**†**^		22 (15.8%)	30 (12.6%)	6 (1.8%)	0/0/0
Bronchopulmonary dysplasia^f^		12 (8.6%)	1 (0.4%)	0 (0%)	0/0/0
Respirator care	Not treated in respirator	72 (51.8%)	210 (87.9%)	339 (99.4%)	0/0/0
	<7 days	51 (36.7%)	26 (10.9%)	2 (0.6%)	
	7 to <14 days	9 (6.5%)	3 (1.3%)	0 (0%)	
	14 or more days	7 (5.0%)	0 (0%)	0 (0%)	
Age at clinical examination (y)^b^^**†**^		23.1 (1.4)	23.2 (1.2)	23.5 (1.1)	0/0/0
Height (cm)	women^b^	162.5 (6.2)	164.5 (5.7)	164.0 (5.8)	0/0/0
	men^b^	178.2 (7.5)	177.7 (6.7)	177.8 (6.9)	0/0/0
BMI (kg/m^2^)^g^	women^b^	24.3 (5.8)	23.7 (4.3)	23.3 (4.3)	0/0/0
	men^b^	24.2 (4.0)	25.3 (4.7)	24.3 (4.3)	0/0/0
Obesity (BMI: 30≥kg/m^2^)	women^b^	12 (16.4%)	9 (7.4%)	17 (9.6%)	0/0/0
	men^b^	8 (12.1%)	18 (15.2%)	8 (4.9%)	0/0/0
Pregnant (currently)^h^^,^^d^		5 (6.8%)	3 (2.5%)	7 (3.9%)	0/0/0
Volume of self-reported leisure-time physical activity (METh/week)^b^		23.3 (13.5)	24.7 (14.5)	25.9 (14.2)	3/5/7

^*^p-value <0.05

^**^p-value <0.01

^**†**^p-value <0.001

^a^Chi-square

^b^Oneway ANOVA F-value

^c^ Data available for participants from Finnish Medical Birth Register (FMBR), participants born 1987–1989

^d^ Fisher’s exact test

^e^Source cohort: FMBR, Finnish Medical Birth Register, participants born 1987–1989; NFBC, Northern Finland Birth Cohort, participants born 1985–1986

^f^Definition based on pediatrician’s diagnoses (for NFBC), and the receipt of supplementary oxygen at 28 days of age (for FMBR) [24].

^g^BMI, body mass index

^h^Of women only

#### Multiple linear and logistic regression models

In Model 1 or the basic model, we controlled for sex, age at the time of the clinical examination, and source cohort (Northern Finland Birth Cohort or Finnish Medical Birth Register). In Model 2, we further adjusted for highest parental education and maternal smoking during pregnancy, which also served as an indicator of family socio-economic status [31], as well as other intrauterine exposures such as maternal hypertensive disorders in pregnancy, maternal gestational diabetes (GDM), and birth weight z-score, and current characteristics such as height, Body Mass Index (BMI, indicator of net nutrition), smoking habit, and self-reported physical activity.

To further examine the relationship between gestational age and lung function, we present the linear regression analyses using finer gestational age groups (GA <28 weeks, 28-<32, 32-<34, 34-<36 and 36-<37 weeks, compared with term-born controls) in S1 Fig. We also assessed the linearity of association between gestational age and adult lung by repeating multilinear regression analyses using gestational age as a continuous variable *(GA)*, as well as its quadratic term (*GA*^*2*^).

### Post hoc and sensitivity analyses

We performed sensitivity analyses in order to test for the effect of gestational age on lung function without known fetal or neonatal causes of lower lung function. The following exclusions were applied in multiple linear regression analyses: (A) BPD, (B) small-for-gestational age (SGA): SD<-2 [20], (C) those who received respirator care (any length), (D) multiple pregnancy, (E) maternal smoking during pregnancy as an indicator of socio-economic status (SES) (S3 Table).

Additionally, we explored the effect of obstructive airways disease on lung function within gestational age groups by comparing lung function among those with or without obstructive airways disease using the logistic regression method. As previously described [12], we formed a composite binary variable to indicate the presence of obstructive pulmonary disease, coded as “yes,” if one or more of the following four signs of obstructive lung disease were present: a history of physician-diagnosed asthma, an entitlement for special reimbursement for asthma medication (self-reported), current use of inhalable glucocorticoid, or a positive bronchodilation test during the clinical examination. We also tested for the risk of positive bronchodilation test among the three GA-groups using logistic regression. The individual effect of covariates applied in multilinear regression models on lung function was also tested. The Ethics Committee of the Helsinki and Uusimaa Hospital District approved the study protocol. Informed written consent was obtained from all participants. The consent protocol was approved by the Ethics Committee of the Helsinki and Uusimaa Hospital District.

## Results

Participants’ background details are provided in Table 1. Mean lung function values (S2 Table) for early and late preterm-born subjects and term-born controls as absolute and percent of predicted were calculated, as well as z-scores based on the global lung standards. Pre-bronchodilator FVC, FEV1, FEV1/FVC, FEF_25%-75%_, FEF_75%_ were all lower among subjects born early preterm than among controls.

We ran multi-variable linear regression models to examine the impact of a range of covariates on the difference in adult lung function between early and late preterm groups compared with controls (Table 2) as well as using finer GA-subgroups (S1 Fig).

**Table 2:** Multiple linear regression: mean difference (95% confidence interval) in lung function z-scores from full-term controls.

Variable	Model	n	Mean difference from term			
			Early Preterm GA<34 weeks		Late preterm GA34-<37 weeks	
			Mean diff	95% CI	Mean diff	95% CI
**zFVC**	1	718	-0.23	-0.40, -0.05**	-0.02	-0.17, 0.13
	2	701	-0.17	-0.35, 0.02	0.02	-0.13, 0.18
**zFEV1**	1	718	-0.44	-0.64, -0.25^†^	-0.12	-0.29, 0.04
	2	701	-0.36	-0.57, -0.16**	-0.06	-0.23, 0.11
**zFEV/FVC**	1	718	-0.29	-0.47, -0.10**	-0.13	-0.29, 0.02
	2	701	-0.26	-0.45, -0.06**	-0.11	-0.27, 0.06
**zFEF**_**75%**_	1	718	-0.34	-0.52, -0,15**	-0.09	-0.25, 0.06
	2	701	-0.29	-0.48, -0.09*	-0.06	-0.22, 0.10
**zFEF**_**25-75%**_	1	718	-0.93	-1.41, -0.46^†^	-0.34	-0.74, 0.05
	2	701	-0.83	-1.33, -0.33**	-0.25	-0.66, 0.17

^*^p-value <0.05

^**^p-value <0.01

^**†**^p-value <0.001

Models applied in multiple linear regression modelling

1. Age, sex, and cohort^a^

2. Model 1 + highest parental education and maternal smoking during pregnancy, maternal pregnancy disorders (gestational hypertension and chronic hypertension, pre-eclampsia and super-imposed pre-eclampsia, gestational diabetes) and birth weight z-score, height, BMI (an indicator of net nutrition), smoking habit, self-reported physical activity

^a^ Source Cohort: FMBR, Finnish Medical Birth Register, participants born 1987–1989; NFBC, Northern Finland Birth Cohort, participants born 1985–1986.

Regression model 1 (Table 2), (adjusting for age, sex, and source cohort), showed poorer pulmonary function among those born early preterm, both in terms of vital capacity and airflow obstruction. Those born late preterm had airflow obstruction, although the differences with term did not attain statistical significance. Model 2 showed that inclusion of parental SES in terms of highest educational attainment and maternal smoking during gestation, maternal gestational disorders, birth weight z-score, current participant characteristics including height, BMI, physical activity, and smoking habit slightly attenuated the associations between preterm birth and vital capacity (FVC), but had minimum impact on the associations with airway obstruction (zFEV/FVC-ratio).

We also assessed the proportion of participants whose FVC, FEV1 or FEV1/FVC z-score was below -1.645 SD (corresponding to 5^th^ percentile). This is shown in Table 3. Participants born early preterm had 3.14-fold odds for low zFEV1 and 2.78-fold odds for low zFEV1/FVC. The associations were slightly attenuated but remained statistically significant when adjusted for Model 2 covariates.

**Table 3:** Numbers and proportions of participants with abnormal lung function (zFVC, zFEV1 or zFEV1/FVC below -1.645 SD) in participants born early and late preterm, compared with controls, with odds ratios and 95% confidence intervals.

	Model	Early preterm (<34 weeks)		Late preterm (34-<37 weeks)		Control (≥37 weeks)
		N (%)	OR (95% CI)	N (%)	OR (95% CI)	N (%)
**zFVC**	1	7 (5.0%)	2.26 (0.78, 6.52)	5 (2.1%)	0.90 (0.29, 2.81)	8 (2.4%)
	2		2.30 (0.65, 8.10)		1.07 (0.31, 3.66)	
**zFEV1**	1	18 (7.5%)	3.14 (1.50, 6.61)	13 (5.4%)	1.29 (0.59, 2.82)	14 (4.1%)
	2		2.65 (1.14, 6.15)		1.35 (0.58, 3.18)	
**zFEV1/FVC**	1	23 (17.2%)	2.78 (1.46, 5.27)	21 (8.8%)	1.41 (0.75, 2.66)	21 (6.2%)
	2		2.34 (1.18, 4.63)		1.30 (0.67, 2.52)	

Models applied in multiple logistic regression modelling

1. Age, sex, and cohort^a^

2. Model 1 + highest parental education and maternal smoking during pregnancy, maternal pregnancy disorders (gestational hypertension and chronic hypertension, pre-eclampsia and super-imposed pre-eclampsia, gestational diabetes) and birth weight z-score, height, BMI (an indicator of net nutrition), smoking habit, self-reported physical activity

^a^ Source Cohort: FMBR, Finnish Medical Birth Register, participants born 1987–1989; NFBC, Northern Finland Birth Cohort, participants born 1985–1986.

### Finer GA sub-groups and GA as continuous variable

We also assessed possible non-linear relationships by including both quadratic and linear terms of gestational age in regression Model 1. The quadratic term was statistically significant for zFEV1/FVC but not for zFVC or zFEV1 (S1 Fig), indicating non-linear association between airflow and gestational age.

To illustrate this non-linearity, we performed a post-hoc analysis using finer GA subgrouping in weeks (<28 (n = 9), 28-<32 (n = 49), 32-<34 (n = 81), 34-<36 (n = 110), and 36-<37 (n = 129) versus term-born controls n = 341)), for the three key lung function outcomes: zFVC, zFEV1, and zFEV1/FVC. The mean differences from controls of these finer gestational age groups are provided in S1 Fig. Broadly, the lower the gestational age, the lower the zFEV1 and zFEV1/FVC. However, much of this relationship was due to the substantially lower zFEV1 and zFEV1/FVC among those born at less than 28 weeks of gestation.

### Lung health and obstructive airways disease

S2 Table describes a range of lung health indicators: physician-diagnosed asthma, asthma medication usage, positive bronchodilation test, history of an obstructive airways disease, smoking and physical activity for the early and late preterm-born subjects and full-term controls. Airway obstruction, fulfilling the criteria to perform bronchodilation test [25,27,30] was more common among the early preterm group, with an inverse association with gestational age. Of the early preterm group 16.5% and of the late preterm group 13.4% had been diagnosed with asthma by a physician. The odds ratio (OR) for having a positive bronchodilation test for those born early preterm was 3.85 (95% confidence interval (CI)): 1.17, 12.67, adjusted for age, sex, and source cohort), compared with the term controls. Adjusted for variables in model 2, it was 2.69 (0.71, 10.12). For the late preterm group, the risk of positive bronchodilation test was not significantly raised (OR: 2.11; 95% CI: 0.65, 6.78). Risk of obstructive airways disease (criteria as described in Table 2) was not raised among either of the preterm groups: early preterm OR was 1.34 (95% CI: 0.80, 2.23), and late preterm OR was 0.94 (95% CI: 0.60, 1.49).

Independent univariate associations between lung function and individual covariates adjusted for in the regression models are provided in the supplementary material (S4 Table).

### Prenatal and neonatal characteristics

Ninety-eight (98) infants (13.6%) had received respirator care (Table 1); the frequency of ventilator treatment within narrower GA groups was as follows: 88.9% (8/9) among extremely preterm (those born before GA<28 weeks), 61.2% (30/49) among very preterm (GA 28-<32 weeks), 35.8% (29/81) among moderately preterm (GA 32-<34 weeks), 19.0% (21/110) among GA 34-<36 weeks, 6.7% (8/119) among GA 36<37 weeks, and 0.6% (2/341) among term-born participants. Analysis of individual covariate associations with lung function parameters (S4 Table) shows that those who had been treated in a respirator had poorer zFEV1/FVC, compared with those who had not. This difference was stronger for those with a longer respirator treatment; for those treated for 14 days or more, the difference was also seen in zFEV1. In our study, thirteen participants fulfilled the criteria for BPD. The prevalence of BPD was 44.4% (4/9) among extremely, 14.3% (7/49) among very, 1.2% (1/81) among moderately and 0.4% (1/239) among late preterm participants.

We assessed the effect of other prenatal and perinatal factors on adult lung function by applying the two regression models in the sensitivity analyses (S3 Table), where we excluded the following: (A) participants with BPD, (B) those born SGA, (C) those who received ventilator treatment (any length), (D) those born of multiple pregnancy, and (E) those exposed to maternal smoking in utero. An exclusion of participants with BPD had no effect on the differences between preterm and term groups; similarly, the exclusion of SGA participants had no impact. An exclusion of those who received respirator care (any length) increased the difference in zFVC and attenuated the difference in zFEV1/FVC between those born early or late preterm, and full-term controls. An exlusion of those from multiple births attenuated the mean difference from controls for zFVC observed among those born early preterm to non-significant. Exclusion of those exposed to maternal tobacco smoking in-utero did not change the results; however, individually tested maternal smoking was an independent predictor of significant airway obstruction (S4 Table). Maternal glucocorticoid treatment (Table 1) was unrelated to lung function.

## Discussion

Our aim was to study the effects of preterm birth on lung function and respiratory health in adulthood, incorporating the whole range of preterm gestational ages. Our results indicate an association between the degree of prematurity and airflow impairment in adult life. The strongest associated was detected among those born most immature. This association between gestational age and lung function was not attentuated by adjusting for underlying risk factors of preterm birth such as maternal pregnancy disorders, maternal smoking during pregnancy, altered fetal growth, or parental socio-economic status. However, maternal smoking during pregnancy on its own was a significant predictor of poorer adult lung function.

Our study is in agreement with previous findings [3–4, 6–8] suggesting that adverse impact of preterm birth on lung function can be detected in adulthood. The results are also in line with the recent meta-analysis of studies mostly on those born very or extremely preterm or at very or extremely low birth weight [5], as well the recent study of young adults born at very low birth weight (VLBW) in Southern Finland [12]. Together with our data, these studies suggest an inverse non-linear relationship between shorter length of gestation and increased risk of obstructive airflow, most prominent among those born most immature, but also observed in those born more mature. This may be an indication of a high degree of complexity of factors contributing to long-term lung health outcomes.

We performed sensitivity analyses excluding those who had received ventilator treatment. Our study shows that despite exclusions, poorer lung functions persisted and the effect was even stronger among those born early preterm. Previous studies have reported poorer long-term lung function among individuals who were born preterm but did not have BPD or any other severe condition that may impact lung function during the peri- or neonatal period: these individuals are often viewed as healthy in terms of pulmonary function [32]. These ‘apparently healthy’ individuals born at earlier gestational weeks may actually represent a particular risk group with poorer lung function due to the lack of receipt of early-life interventions. Our data showed an inverse, partly non-linear relationship between lung function and gestational age (S1 Fig) which could partially reflect the differences in early-life treatment. Furthermore, environmental exposures such as maternal smoking during pregnancy, and lower SES status (education as an indicator) could modify the association [4,15–18].

So far, only few studies have assessed lung function across the whole range of gestational ages. A longitudinal ALSPAC cohort study of 8-9-year olds reported reductions in zFEV1 of -0.49 among those born at 25–32 weeks of gestation, -0.49 among those born at 33–34 weeks, and 0.01 among those born at 35–36 weeks, compared with those born at term [8]. These results are similar to our observations among young adults in Finland. Interestingly, in the ALSPAC the mean zFEV1 differences compared with control were undetectable later on, at the age of 14–17 years [8].

Our findings do not indicate the mechanism of airflow impairment. The most significant contributor to airflow rate is resistance to airflow, which is primarily influenced by airway diameter [33]. While airway size and lung volume/size grow in parallel, this growth may not always be proportional and may result in smaller airways in relation to lung volume, a phenomenon referred to as dysanapsis [34]. Recent studies using proxy measures of dysanapsis derived from spirometry suggest that dysanapsis may be a key contributor to airflow impairment in children and adults born very preterm [35, 36] and that smallest airways in relation to lung volume are seen in very preterm adults with a history of BPD [35]. Spirometry-derived indices may, however, underestimate the extent of impairment in lung function. More direct measurements of ventilation inhomogeneity obtained through inert tracer gas washout tests are consistent with impaired ventilation distribution in the more proximal conducting airways in children born very preterm [37].

While our findings were largely independent of pregnancy conditions underlying preterm birth, maternal smoking during pregnancy was an independent predictor of airway obstruction. This is consistent with a recent Finnish study of adults born preterm at VLBW [12]. Although we lacked data on childhood tobacco exposure, a study of Russian children aged 8–12 years found that asthma was more strongly predicted by fetal than postnatal tobacco smoke exposure [38]. Participants who were current or former daily smokers had more airway obstruction, but smoking habit did not explain the association between preterm birth and the airway obstruction.

Our findings indicate the risks for obstructive airways disease (See Methods for criteria), among those born early preterm (OR: 1.34, 95% CI: 0.81, 2.22) and late preterm (OR: 0.96, 95% CI 0.61, 1.51) are comparable with a meta-analysis reporting a random-effects (OR: 1.19, 95% CI 0.94, 1.51) for subjects aged over 10 years with substantial heterogeneity [7]. A Scandinavian register study reported a risk ratio of 1.05, 95% CI 1.04, 1.06) for childhood hospitalization for asthma for each week’s decrease in gestational age compared with those who were term-born (GA 39-<42 weeks) [18]; this association became steeper at earlier gestational ages. A Swedish register study using asthma medication purchases as an outcome suggested that the relationship between preterm birth and asthma medication could largely be explained by higher rates of asthma medication use among those born extremely preterm [39]. A Finnish hospital and national register study used special reimbursement for asthma medication as an outcome; this was based on predefined clinical criteria specified in a physician’s statement and thus was more accurate than medication purchase only. The study showed increased rates of asthma medication at all degrees of prematurity, but again the association with gestational age became steeper at lower gestational ages [4]. A study among 31-year-olds born in Northern Finland reported an OR: 1.14 (95% CI 0.92, 1.40) for self-reported asthma for GA less than 36 weeks versus GA over 36 weeks [40]. Taken together, these modest associatons are consistent with a weak inverse relationship between earlier gestational age and higher asthma prevalence; this relationship may be stronger at lower gestational ages. It is also of note that obstructive airways disease among individuals born very or extremely preterm may exhibit a reduced response to asthma medication and not always qualify for asthma diagnosis [41,42].

Only a few studies have followed up pulmonary impact of preterm birth to old age. A Swedish register study of the 1925–1949 birth cohort found that hospital-treated chronic obstructive pulmonary disease (COPD) was predicted both by preterm birth and intrauterine growth restriction, whereas hospital-treated asthma was predicted by preterm birth only—however, only among women [43]. The Helsinki 1934–1944 Birth Cohort Study reported that special reimbursement for obstructive lung disease medication was predicted by slow intrauterine growth, but not by preterm birth, albeit the number of preterm-born subjects was low [44]. In addition, a meta-analysis has linked lower birth weight with lower FEV1 throughout adult-life [45]. While differences in study designs make it difficult to draw clear conclusions, adult studies are overall consistent in reporting prenatal origins of obstructive lung disease.

Our participants are in their early twenties and at the age of maximum lung function. Even with normal natural decline in lung function, those with lower peak function are expected to reach symptomatic levels earlier. Moreover, this decline may be accelerated in those with observable airway obstruction in adulthood. The CARDIA cohort study performed spirometry among participants at 18–30 years of age and again at 35–50 years. Drops of over 5% in FEV1 and FVC were observed in those with airway obstruction at baseline, while less than 3% in those with normal baseline lung function [46]. It is essential to explore whether this occurs in adults born preterm and to focus on follow-up studies of present cohorts of adults born preterm, where currently the oldest members of cohorts are approaching their 40s.

In terms of prevention, it is important to slow down the inevitable deterioration of lung function [10] and the development of symptomatic obstructive airways disease for those at heightened risk [47,48], such as those born early preterm, including those with no apparent pulmonary problems during peri- and neonatal periods. While there are no studies suggesting effective methods of prevention with a focus on adults born preterm, abstinence from smoking is obvious. Promotion of physical activity may not prevent obstructive airway disease *per se*. However, adults born preterm undertake less physical activity than those born at term [49], which may in part be a consequence of airway obstruction. Promotion of health-enhancing physical activity can be recommended as it has many other health benefits.

### Limitations

While we have extensive prenatal and neonatal data, we lack data on childhood exposures, such as respiratory infections, that may predispose to chronic airways obstruction later on.

Different diagnostic criteria for BPD were applied in our two source cohorts; among those recruited from the FMBR we were able to independently confirm the diagnosis by assessing the need of supplementary oxygen at 28 postnatal days from medical records, while in the NFBC the diagnosis was made by a clinician and we had no access to the individual criteria of diagnosis. It is therefore possible that we are missing a few NFBC individuals who would have had BPD according to the criteria we used for FMBR participants. Furthermore, treatment of preterm-born infants today differs from that of our participants who were born during the pre-surfactant era, when antenatal glucocorticoid treatment was only being introduced in Finland. Data on antenatal glucocorticoid treatment was only available for participants from the Finnish Medical Birth Registry born between1987 and 1989; 22.2% of those born early preterm had been exposed to the treatment. However, these limitations are relevant for individuals born very or extremely preterm and thus less pertinent for our study, which was designed to assess the whole spectrum of preterm birth and accordingly also had limited power to assess those born very or extremely preterm.

Further, we have no data on parental respiratory health or atopy and cannot exclude confounding by these factors.

## Conclusion

Our study shows that adults born early preterm have airflow limitation. This association seems to be independent of underlying pregnancy disorders or risk factors, such as maternal gestational smoking or socio-economic position. Our study showed an inverse association between gestational age and obstructive airflow, much of which was due to those born most immature. In addition, adults born early preterm had slightly lower vital capacity, which may be partly explained by intrauterine growth restriction, which commonly affects this group. Most differences between those born late preterm and controls were not statistically significant. Our results are consistent with previous studies reporting an inverse relationship between the degree of prematurity and airflow obstruction.

Obstructive airways disease may in part have its origins in preterm birth and prenatal life. We encourage healthcare professionals to be vigilant when dealing with individuals born at early gestational weeks even with no other significant health conditions impacting pulmonary development. These individuals represent a potential unacknowledged risk group for poor long-term lung health outcomes.

## Supporting information

S1 Fig The error bars show mean differences (95% confidence intervals) with controls in each gestational age group. Black line indicates zero difference from control.
(TIF)

S1 Table Description of variables and their subgroupings. The ESTER birth cohort (births in Northern Finland during 1985–89); clinical examination during 2009–11.
(DOCX)

S2 Table Univariate comparisons of spirometry data and lung health between gestational age groups. (DOCX)

S3 Table Mean difference (95% CI) in lung function z-scores, compared with full-term controls: sensitivity analyses. (DOCX)

S4 Table Individual effect of each covariate association with lung function as zFVC, zFEV1, and zFEV1/FVC. (DOCX)
