# Supplementary figures and images for: Lung function in adults born preterm
Source: PLoS One. 2018 Oct 19;13(10):e0205979. doi: 10.1371/journal.pone.0205979 (PMC6195283; doi:10.1371/journal.pone.0205979)

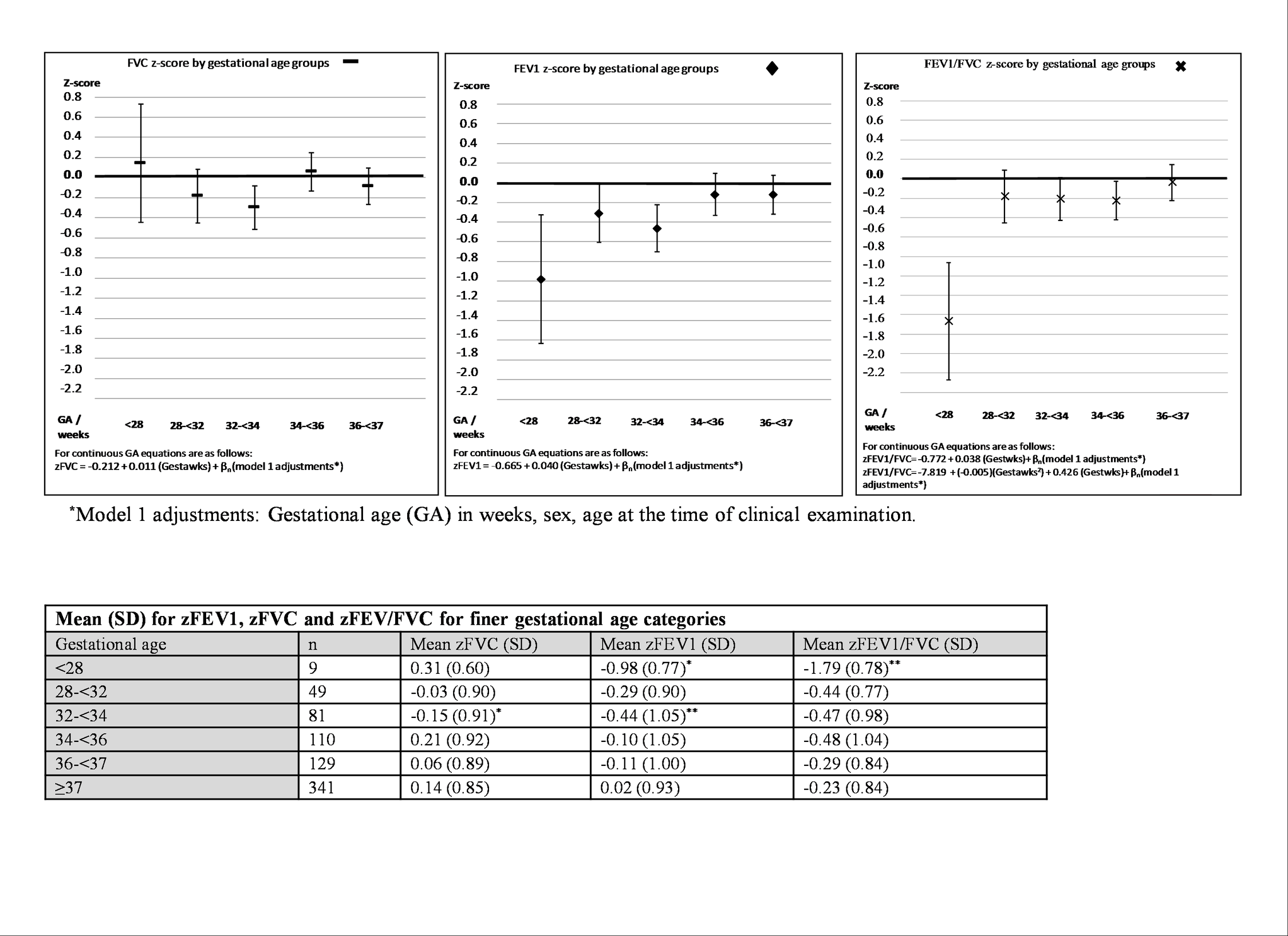

Supplement: S1 Fig — Black line indicates zero difference from control. (TIF) [file pone.0205979.s001.tif]
