# Supplementary material for: Lung function in adults born preterm
Source: PLoS One. 2018 Oct 19;13(10):e0205979. doi: 10.1371/journal.pone.0205979 (PMC6195283; doi:10.1371/journal.pone.0205979)
Supplement: S1 Table — The ESTER birth cohort (births in Northern Finland during 1985–89); clinical examination during 2009–11. (DOCX) [file pone.0205979.s002.docx]

**S1 Table.** **Description of variables and their subgrouping. The ESTER birth cohort (births in Northern Finland during 1985-89): clinical examination during 2009-11.**

**Age:** The risk of asthma may be different at different stages of childhood and adulthood. We therefore adjusted for age at the time of the clinical examination in order to account for age-specific risk of asthma.

**Sex:** Male vs. female. Male sex has been associated with a greater risk of asthma and atopic disease.

**Source cohort:** Participants were selected from the Northern Finland Birth Cohort (NFBC) for the years 1985-1986 and from the Finnish Medical Birth Register (FMBR) for the years 1987-1989. The main effect of source cohort was controlled for in all analyses.

**Parental education:** Participants reported the educational attainment of their parents, and the attainment of the higher-educated parent served as an indicator of family socio-economic status. Education was classified into 3-categories: secondary or less (including unknown education), lower tertiary, and upper tertiary education. Categorical data was coded as dummy variables. Socioeconomic differences in asthma risk have been reported (highest risk among low SES families).

***Intrauterine exposures:***

**Maternal smoking during gestation (No/Yes)** obtained from birth records [19]. Fetal exposure to tobacco smoke has been associated with poorer growth and poorer lung function in children.

**Maternal disorders in pregnancy:**

- **Maternal hypertensive disorders of pregnancy** were independently confirmed according to prevailing criteria by reviewing original hospital records [20] and grouped into the following 3 groups: 1) **gestational hypertension or chronic hypertension**, 2) **pre-eclampsia, including super-imposed pre-eclampsia,** 3) **normotensive** (including cases with insufficient data to assess diagnosis) and treated as dummy variables. Cases with proteinuria were coded as normotensive if no increase in the blood pressure was observed.
- **Maternal gestational diabetes (GDM)** (No/Yes) was confirmed according to prevailing criteria by reviewing original hospital records as described [21]. GDM has been associated with poorer health outcomes, in particular higher risk of cardiovascular diseases and hypertension in later life. This early life environment is likely to impact later pulmonary function.

**Birth weight z-scores** were calculated relative to gestational age and sex using Finnish contemporary growth standards [22]: -2SD was used as a cut-off point for small- for-gestational- age (SGA). Poorer fetal growth has been associated with small lungs and poorer lung function.

***Participant health:***

**Obstructive airways disease** was explored using the following variables:

a) **Physician-diagnosed asthma (No/Yes):** response to the question: ‘Have you ever been diagnosed with asthma by a physician?’

b) **History of obstructive airways disease (No/Yes):** A composite binary variable indicating obstructive pulmonary disease was present if one or more of the following four signs of obstructive lung disease were present: a history of physician-diagnosed asthma, an entitlement for special reimbursement for asthma medication (self-reported), current use of inhalable glucocorticoid, or a positive bronchodilation test during the clinical examination.

**Smoking habit** was reported via a questionnaire in conjunction with the clinical examination and individuals were classified as: a daily smoker, former smoker (smoking occasionally up to once a week or had formerly smoked but had permanently given up) or a never smoker (having tried previously, never smoked or data was missing). Smoking has an adverse effect on lung function.

**Height:** Participant height was measured during the clinical examination. Taller stature has been associated with larger pulmonary volume ‘forced vital capacity’ (FVC) and better lung function.

**Body mass index** was calculated using height (m) and weight (kg) measured during the clinical examination. Obesity has been associated with poorer lung function.

**Self-reported leisure-time physical activity** was recorded as hours spent per week engaged in physical activity assessed by questionnaire, expressed as metabolic equivalent-hours per week (METh/week). A higher level of physical activity has been associated with better pulmonary function (FVC and forced expiratory volume in 1 second (FEV1)).

**Missing data:** For a small number of participants, data for maternal pre-pregnancy BMI, hypertension in pregnancy, or the participant’s physical activity was missing. The number of participants with missing data for these variables is shown in Table 1. Pre-pregnancy BMI was not used as a covariate. For hypertension in pregnancy, we classified those with missing data in the normotensive category. For physical activity participants with missing physical activity data were coded as missing and excluded from the analyses.
