# Supplementary material for: Lung function in adults born preterm
Source: PLoS One. 2018 Oct 19;13(10):e0205979. doi: 10.1371/journal.pone.0205979 (PMC6195283; doi:10.1371/journal.pone.0205979)
Supplement: S2 Table — (DOCX) [file pone.0205979.s003.docx]

**S2 Table. Univariate comparisons of spirometry data and lung health between gestational age groups.**

| **Lung health variable** | | **Early preterm (<34 weeks )** | **Late preterm (34-<37 weeks)** | **Control (≥37 weeks)** |
| --- | --- | --- | --- | --- |
|  |  | n=139 | n=239 | n=341 |
|  |  | **mean (SD)/ n(%)** | **mean (SD)/ n(%)** | **mean (SD)/ n(%)** |
| FVC, L^a^ | | 4.6 (1.2) | 4.8 (1.1) | 4.8 (1.0) |
| FEV1, L^a^ | | 3.8 (0.9) | 4.0 (0.8) | 4.0 (0.8)**^*^** |
| FEV1/FVC, %^a^ | | 82.0 (7.1) | 83.1 (6.9) | 84.1 (5.7)**^†^** |
| FEF_25-75%_,L/s^a^ | | 3.9 (1.2) | 4.2 (1.3) | 4.4 (1.2) |
| FEF_75%_, L^a^ | | 7.1 (1.9) | 7.6 (2.0) | 7.8 (1.9) |
| FEF_50%_, L^a^ | | 4.5 (1.4) | 4.9 (1.5) | 5.1 (1.4)**^**^** |
| zFVC z-score^a^ | | -0.08 (0.89) | 0.13 (0.91) | 0.14 (0.85)**^*^** |
| zFEV1 z-score^a^ | | -0.43 (0.99) | -0.10 (1.02) | 0.02 (0.93)**^†^** |
| zFEV1/FVC z-score^a^ | | -0.55 (0.95) | -0.38 (0.99) | -0.23 (0.84)**^†^** |
| zFEF_75%_ z-score^a^ | | -0.20 (0.93) | 0.06 (0.97) | 0.18 (0.89)**^†^** |
| zFEF_25-75%_ z-score^a^ | | 0.00 (2.30) | 0.59 (2.50) | 0.95 (2.30)**^†^** |
| zFVC z-score<-1.645SD^b^ | | 7 (5.0%) | 5 (2.1%) | 8 (2.3%) |
| zFEV1 z-score <-1.645SD^b^ | | 18 (7.5%) | 13 (5.4%) | 14 (4.1%)**^**^** |
| zFEV1/FVC z-score <-1.645SD^c^ | | 23 (17.2%) | 21 (8.8%) | 21 (6.2%)**^*^** |
| FVC,% of predicted^a^ | | 97.4 (11.7) | 99.9 (11.2) | 99.8 (10.4) |
| FEV1. % of predicted^a^ | | 90.3 (11.6) | 94.0 (11.7) | 95.2 (10.4)**^†^** |
| FEV1/FVC. % of predicted^a^ | | 93.2 (7.7) | 94.5 (7.8) | 96.0 (6.4)**^†^** |
| PEF. % of predicted^a^ | | 87.4 (11.8) | 91.2 (14.3) | 93.3 (12.3)**^†^** |
| FEF_50%._ % of predicted^a^ | | 76.9 (20.6) | 82.8 (23.4) | 86.2 (20.7)**^†^** |
| Completed bronchodilation test^c^ | | 59 (42.4%) | 78 (32.6%) | 94 (27.6%)**^†^** |
| Positive bronchodilation test^c,d^ | | 7 (5.0%) | 7 (2.9%) | 5 (1.5%) |
| Positive bronchodilation test among those with FEV1/FVC <1.645SD^b^ | | 7 (30.4%) | 4 (19.0%) | 3(14.2%) |
| Current inhaled glucocorticoids | | 5 (3.6%) | 2 (0.8%) | 7 (2.1%) |
| Asthma ever diagnosed by a physician ^c^ | | 23 (16.5%) | 32 (13.4%) | 49 (14.4%) |
| History of obstructive airways disease^c,e^ | | 29 (20.9%) | 38 (15.9%) | 56 (16.5%) |
| Smoking habit ^c^ | Never smoker (incl missing) | 89 (64.0%) | 157 (65.7%) | 229 (67.2%) |
|  | Former Smoker | 13 (9.4%) | 30 (12.6%) | 40 (11.7%) |
|  | Daily smoker | 37 (26.6%) | 52 (21.8%) | 72 (21.1%) |
| Current antihistamine use^c^ | | 11 (7.9%) | 9 (3.8%) | 14 (4.1%) |

**^*^**p-value <0.05

**^**^**p-value <0.01

**^†^**p-value <0.001

^a^Oneway Anova

^b^Fisher’s exact test

^c^Chi-square test

^d^Positive bronchodilation test (change in FEV1 or FVC >12%) [30]

^e^Based on a composite obstructive airways disease-outcome variable where a one or more of the following four criteria indicated a history of obstructive airways disease: history of physician-diagnosed asthma, current use of inhalable glucocorticoid, a current receipt of special reimbursement for asthma medication, or positive bronchodilation test at the time of clinical examination.
