# Supplementary material for: Lung function in adults born preterm
Source: PLoS One. 2018 Oct 19;13(10):e0205979. doi: 10.1371/journal.pone.0205979 (PMC6195283; doi:10.1371/journal.pone.0205979)
Supplement: S3 Table — (DOCX) [file pone.0205979.s004.docx]

**S3 Table. Mean difference (95% CI) in lung function z-scores, compared with full-term control: sensitivity analyses.**

**Exclusions:** A) cases from multiple pregnancies, B) cases with Bronchopulmonary Dysplasia (BPD), C) cases exposed to maternal smoking during gestation, D) cases with small-for-gestational age birthweight (less than -2SD), and E) cases treated with ventilator

| **Lung function parameter** | **Model** | **n** | **Mean difference from term (95% CI)** | | | |
| --- | --- | --- | --- | --- | --- | --- |
|  |  |  | **<34wks** | **95% CI** | **34-<37wks** | **95% CI** |
| **No exclusions** | | | | | | |
| **zFVC** | 1 | 718 | -0.23 | -0.40, -0.05^**^ | -0.02 | -0.17, 0.13 |
|  | 2 | 701 | -0.17 | -0.35, 0.02 | 0.02 | -0.13, 0.18 |
| **zFEV1** | 1 | 718 | -0.44 | -0.64, -0.25_†_ | -0.12 | -0.29, 0.04 |
|  | 2 | 701 | -0.36 | -0.57, -0.16^**^ | -0.06 | -0.23, 0.11 |
| **zFEV/FVC** | 1 | 718 | -0.29 | -0.47, -0.10^*^ | -0.13 | -0.29, 0.02 |
|  | 2 | 701 | -0.26 | -0.45, -0.06^*^ | -0.11 | -0.27, 0.06 |
| **zFEF_75%_** | 1 | 718 | -0.34 | -0.52, -0.15^**^ | -0.09 | -0.25, 0.06 |
|  | 2 | 701 | -0.29 | -0.48, -0.09^*^ | -0.06 | -0.22, 0.10 |
| **zFEF_25-75%_** | 1 | 718 | -0.93 | -1.41, -0.46^†^ | -0.34 | -0.74, 0.05 |
|  | 2 | 701 | -0.83 | -1.33, -0.33^**^ | -0.25 | -0.66, 0.17 |
| **Multiple births excluded** | | | | | | |
| zFVC | 1 | 650 | -0.18 | -0.37, 0.02 | -0.03 | -0.18, 0.12 |
|  | 2 | 635 | -0.11 | -0.31, 0.09 | -0.01 | -0.16, 0.15 |
| zFEV1 | 1 | 650 | -0.40 | -0.61, -0.18^†^ | -0.15 | -0.32, 0.02 |
|  | 2 | 635 | -0.30 | -0.53, -0.08**^**^** | -0.09 | -0.27, 0.08 |
| zFEV1/FVC | 1 | 650 | -0.28 | -0.49, -0.08**^**^** | -0.16 | -0.32, -0.00^*^ |
|  | 2 | 635 | -0.25 | -0.46, -0.03**^*^** | -0.11 | -0.27, 0.06 |
| **Exposure to maternal smoking during pregnancy excluded** | | | | | | |
| zFVC | 1 | 595 | -0.23 | -0.43, -0.03^*^ | -0.02 | -0.19, 0.15 |
|  | 2 | 581 | -0.17 | -0.38, 0.04 | 0.01 | -0.16, 0.19 |
| zFEV1 | 1 | 595 | -0.43 | -0.65 , -0.21^†^ | -0.08 | -0.27, 0.10 |
|  | 2 | 581 | -0.35 | -0.59, -0.12**^**^** | -0.04 | -0.24, 0.15 |
| zFEV1/FVC | 1 | 595 | -0.26 | -0.46, -0.06^*^ | -0.07 | -0.23, 0.10 |
|  | 2 | 581 | -0.24 | -0.46, -0.02**^*^** | -0.07 | -0.25, 0.11 |
| **BPD cases excluded** | | | | | | |
| zFVC | 1 | 705 | -0.23 | -0.41, -0.05 | -0.02 | -0.17, 0.13 |
|  | 2 | 688 | -0.18 | -0.36, 0.01 | 0.02 | -0.14, 0.17 |
| zFEV1 | 1 | 705 | -0.42 | -0.62, -0.21 | -0.13 | -0.29, 0.04 |
|  | 2 | 688 | -034 | (-0.55, -0.13) | -0.07 | (-0.24, 0.11) |
| zFEV1/FVC | 1 | 705 | -0.25 | -0.44, -0.06 | -0.13 | -0.28, 0.02 |
|  | 2 | 688 | -0.21 | -0.41, -0.02**^*^** | -0.10 | -0.26, 0.06 |
| **SGA excluded** | | | | | | |
| zFVC | 1 | 660 | -0.20 | -0.39, -0.01^*^ | 0.02 | -0.14, 0.17 |
|  | 2 | 646 | -0.18 | -0.37, 0.01 | 0.03 | -0.13, 0.19 |
| zFEV1 | 1 | 660 | -0.38 | -0.59, -0.18^†^ | -0.06 | -0.23, 0.11 |
|  | 2 | 646 | -0.35 | -0.56, -0.14**^**^** | -0.04 | -0.21, 0.14 |
| zFEV1/FVC | 1 | 660 | -0.24 | -0.43, -0.04^*^ | -0.10 | -0.25, 0.06 |
|  | 2 | 646 | -0.21 | -0.41, -0.01**^*^** | -0.08 | -0.24, 0.08 |
| **Those treated with ventilator excluded** | | | | | | |
| zFVC | 1 | 620 | -0.34 | -0.56, -0.21^**^ | -0.03 | -0.18, 0.11 |
|  | 2 | 606 | -0.28 | -0.51, -0.06^*^ | -0.01 | -0.16, 0.15 |
| zFEV1 | 1 | 620 | -0.41 | -0.65, -0.16^**^ | -0.08 | -0.25, 0.09 |
|  | 2 | 606 | -0.32 | -0.57, -0.07^*^ | -0.03 | -0.21, 0.14 |
| zFEV1/FVC | 1 | 620 | -0.08 | -0.32, 0.14 | -0.05 | -0.20, 0.11 |
|  | 2 | 606 | -0.05 | -0.28, 0.19 | -0.02 | -0.19, 0.14 |

^*^p-value <0.05

^**^p-value <0.01

^†^p-value <0.001

**Models applied in multiple regression modelling:**

1. Age, sex and cohort^a^

2. Model 1 and highest parental education and maternal smoking during pregnancy, pregnancy disorders (gestational hypertension and chronic hypertension, pre-eclampsia and super-imposed pre-eclampsia (excluding proteinuria), gestational diabetes) and birth weight z-score, height, body mass index (an indicator of net nutrition), smoking habit, self-reported physical activity

^a^ Cohort: FMBR, Finnish Medical Birth Register, participants born 1987-1989; NFBC, Northern Finland Birth Cohort, participants born 1985-1986
