# Supplementary material for: Lung function in adults born preterm
Source: PLoS One. 2018 Oct 19;13(10):e0205979. doi: 10.1371/journal.pone.0205979 (PMC6195283; doi:10.1371/journal.pone.0205979)
Supplement: S4 Table — (DOCX) [file pone.0205979.s005.docx]

**S4 Table. Individual effect of each covariate on lung function as zFVC, zFEV1, and zFEV1/FVC.**

**Note: Age, sex and cohort: unadjusted, other associations adjusted for age, sex, cohort.**

| Covariates | | **zFVC** | **95% CI** | **zFEV1** | **95% CI** | **zFEV1/FVC** | **95% CI** |
| --- | --- | --- | --- | --- | --- | --- | --- |
| Age at clinical examination, yr | | 0.00 | -0.07, 0.07 | -0.04 | -0.12, 0.04 | -0.05 | -0.12, 0.03 |
| Sex (REF: men vs. women) | | -0.01 | -0.14, 0.12 | -0.05 | -0.19, 0.10 | -0.04 | -0.18, 0.09 |
| Source cohort (REF: FMBR^a^ vs. NFBC^b^) | | -0.02 | -0.20, 0.16 | 0.16 | -0.04, 0.36 | 0.25 | 0.06, 0.44 |
| Highest parental education (REF: Secondary or less) | Lower tertiary | -0.09 | -0.30, 0.11 | 0.00 | -0.23, 0.23 | 0.15 | -0.07, 0.36 |
|  | Upper tertiary | -0.00 | -0.17, 0.16 | 0.04 | -0.14, 0.22 | 0.08 | -0.09, 0.25 |
| Maternal smoking during pregnancy | | 0.02 | -0.16, 0.19 | -0.11 | -0.31, 0.08 | -0.19 | -0.37, -0.01 |
| Maternal hypertensive disorders (REF: Normotensive) | Gestational or chronic hypertension | -0.03 | -0.22, 0.17 | -0.01 | -0.23, 0.21 | 0.02 | -0.18, 0.23 |
|  | Pre-eclampsia, incl super-imposed pre-eclampsia | -0.22 | -0.43, -0.00 | -0.32 | -0.56, -0.08 | -0.13 | -0.35, 0.10 |
| Maternal gestational diabetes | | -0.11 | -0.50, 0.28 | -0.28 | -0.71, 0.15 | -0.25 | -0.65, 0.16 |
| Birth weight z-score,SD | | 0.10 | 0.05, 0.15 | 0.11 | 0.05, 0.17 | 0.01 | -0.05, 0.06 |
| Height, cm | | 0.01 | -0.00, 0.02 | 0.01 | -0.01, 0.02 | -0.01 | -0.02, 0.01 |
| Respirator care, d^c^† (REF: cases who received no respirator care) | <7 days | 0.21 | -0.02, 0.45 | -0.13 | -0.39, 0.13 | -0.45 | -0.69, -0.22 |
|  | 7 - 13 days | 0.10 | -0.42, 0.62 | -0.30 | -0.88, 0.27 | -0.62 | -1.15, -0.08 |
|  | ≥14 days | -0.09 | -0.77, 0.59 | -0.88 | -1.64, -0.13 | -1.19 | -1.89, -0.50 |
| Body mass index, kg/m^2^ | | 0.01 | -0.00, 0.03 | -0.01 | -0.03, 0.01 | -0.03 | -0.05, -0.02 |
| Smoking habit^c^ (REF: never smoker) | | 0.03 | -0.05, 0.11 | -0.03 | -0.12, 0.06 | -0.09 | -0.17, -0.01 |
| Self-reported leisure-time physical activity, METh/week | | 0.01 | 0.00, 0.01 | 0.01 | 0.00, 0.01 | 0.00 | -0.00, 0.01 |

^a^FMBR, Finnish Medical Birth Register, participants born 1987-1989

^b^NFBC, Northern Finland Birth Cohort, participants born 1985-1986

^c^Smoking-habit: Daily smoker and former were compared against never smokers as a reference group
